# The joint NETTAB/Integrative Bioinformatics 2015 Meeting: aims, topics and outcomes

**DOI:** 10.1186/s12859-017-1532-0

**Published:** 2017-03-23

**Authors:** Paolo Romano, Ralf Hofestädt, Matthias Lange, Domenica D’Elia

**Affiliations:** 1 IRCCS AOU San Martino IST, Genoa, I-16132 Italy; 2 University of Bielefeld, Bielefeld, D-33615 Germany; 3 Leibniz Institute of Plant Genetics and Crop Plant Research (IPK), Gatersleben, D-06466 Germany; 4 Institute for Biomedical Technology – National Research Council, Bari, I-70126 Italy

## Abstract

The 15^th^ International NETTAB workshop and the 11^th^ Integrative Bioinformatics Symposium were held together in Bari, on October 14–16, 2016, as Joint NETTAB/IB 2015 Meeting. A special topic for the meeting was “Bioinformatics for ncRNA”, but the traditional topics of both meetings series were also included in the event.

About 60 scientific contributions were presented, including six keynote lectures, one special guest lecture, and many oral communications and posters. A “Two-Day Hands-on Tutorial” event was organised before the workshop.

Selected full papers from some of the best works presented in Bari were submitted either to the Journal of Integrative Bioinformatics or to a purpose Call for a Supplement of BMC Bioinformatics.

Here, we provide an overview of meeting aims and scope. We also shortly introduce selected papers that have been either accepted for publication in this Supplement or published in the Journal of Integrative Bioinformatics, for a more complete presentation of the outcomes of the meeting.

## NETTAB workshops and integrative bioinformatics symposia

Network Tools and Applications in Biology (NETTAB) Workshops is a series of International meetings held annually in Italy since 2001 with the aim of studying and analysing the impact of innovative Information and Communication Technologies (ICTs) on bioinformatics and biomedical research [1]. Scientific sessions are focused on tools, systems and applications that can be conceived and developed by adopting a given ICT, as well as its expected impact in Life Sciences. They are usually introduced by keynote lectures and completed by selected oral communications. Discussions within oral and poster sessions allow participants to present and discuss their work and ideas on the main workshop topics. NETTAB workshops also includes some tutorials on cutting-edge technologies open to both young and senior researchers. Tutorial theme changes every year in order to cope with on-going technological and scientific innovation in the field.

Integrative Bioinformatics is nowadays widely used in *omics* and Big Data age. The fourth paradigm “Data-Intensive Scientific Discovery” [2] describes the potential of rapidly emerging field of data-intensive science for scientific breakthroughs. This became evident for Life Sciences quite early and it was the driving force to establish the series of symposia “Integrative Bioinformatics” (IB), which was jointly built up, more than 10 years ago, by the research groups Bioinformatics/Medical Informatics, at the University of Bielefeld, and Bioinformatics, IPK-Gatersleben. The aim was to establish synergies between computer science, biology and bioinformatics as border breaking communities towards an integrated knowledge discovery process.

Both young researchers and senior scientists from biology, medicine and computer science, annually meet to discuss applied technology and recent scientific challenges. To make the IB symposia attractive for many researchers world-wide, all submissions are peer-reviewed for publication in the Journal of Integrative Bioinformatics (JIB), an open access journal publishing original peer-reviewed research articles in all aspects of integrative bioinformatics [3]. Works accepted for publication are introduced in the event programme as presentations. A further decision was to bring the workshop to important research hot spots, such as Hangzhou (China) in 2012, Cambridge (United Kingdom) in 2010 and Ghent (Belgium) in 2007, and attract scientists in place. With this concept, IB attracted dozens of researchers every year and set the seed for a number of research projects and collaborations.

## NETTAB & IB 2015: the joint meeting

### Scope and rationale

In 2015, the NETTAB and Integrative Bioinformatics initiatives joined their forces for a synergistic event able to attract more researchers than each event did in the past on its traditional topics. The Joint NETTAB and Integrative Bioinformatics 2015 Meeting (NETTAB/IB 2015), was held on October 14–16, 2015, in Bari, Italy [4].

The rationale of the meeting was drawn from the basic fact that high throughput “*omics*” technologies generate large quantities of high dimensional data, while biological data are scattered across thousands of biological databases and hundreds of scientific journals. In this context, the challenge for Integrative Bioinformatics is to capture, model, integrate and analyse these data in order to provide systematic insights into complex biological systems. At the same time, the huge size of these data make it necessary to deploy the vast majority of retrieval and analysis tasks over the Internet. Network tools, which are the scope of NETTAB workshops, are then fundamental to achieve the above goals. Improved user interfaces may allow researchers a more productive activity on the web.

The joint event has provided an excellent environment and a range of opportunities to present and discuss methods, theoretical approaches, algorithms, tools, platforms, practical applications and experiences. More than 70 participants from 14 European countries met in the capital of Apulia to discuss approaches in integrative bioinformatics and present recent progresses in the topics scope. The special NETTAB/IB 2015 topic was “Bioinformatics for ncRNA”. The call for contributions in this research domain was launched for the presentation of bioinformatics methods, algorithms, tools and applications for the analysis of non-coding RNAs (ncRNAs) role in gene expression and genome maintenance and included: i) NGS data analysis tools, pipeline and integrated platforms; ii) computational approaches and software for the prediction of ncRNA structures and functions; iii) tools and computational methods for the prediction of ncRNA functional interactions and regulatory networks; iv) databases and web-based integrated analysis tools.

The traditional topics of both Integrative Bioinformatics Symposia and NETTAB workshops were also included. For a full list of topics, see the meeting web site [4].

### Scientific programme

The programme included six keynote lectures, as well as one special guest lecture and 18 oral communications. Keynote lectures given by Paul Kersey, Emek Demir, and Artemis Hatzigeorgiou had a clear emphasis on the main scientific topics of the meeting.

Paul Kersey, currently at the European Bioinformatics Institute as leader of the non-vertebrate genomics group, gave a talk on “Adventures in Cereal Genomics”. Cereal genomes are especially large and difficult to assemble, but are nonetheless increasingly available, including draft assemblies for the hexaploid genome of bread wheat. In his talk, he discussed the challenges in data management presented by large cereal genomes, demonstrated some solutions and exposed what can be learnt through their large-scale comparative analysis.

Emek Demir is Assistant Professor at the Oregon Health & Science University (OHSU). He has been in charge of the Pathway Information Resource for many years. Advances in molecular technologies have led to the generation of data and information about cellular processes at an unprecedented increasing rate. Current means of knowledge representation cannot cope with the complexity and volume of new generated data. The Pathway Commons project aims to create a common language and platform for building cell maps, defined as system level, integrated models of cellular processes. Demir’s talk, “Building Cell Maps”, reported on four applications of cell maps aimed at i) finding causal explanations to correlations in large scale high throughput tumor profiles; ii) improving network inference algorithms; iii) finding metabolic tumor vulnerabilities; iv) finding mutually altered pathway fragments.

Artemis Hatzigeorgiou is Principal Investigator at the B.S.R.C. “Alexander Fleming”, adjunct assistant Professor at the University of Pennsylvania and co-founder of Synaptic, Ltd, a computer science company located at Herakleion, Crete (GR). She developed DIANA-microT, one of the first published microRNA target prediction software, and presently leads the DIANA Lab providing algorithms, databases and analysis software for the analysis of ncRNAs. In her talk “Characterising miRNA promoters and miRNA targets”, she provided an overview on the biological significance of miRNAs and reported about most recent advances on their functional interaction with long ncRNAs, currently at the centre of the biological research because of their involvement in many important biological processes and human diseases. In this specific field, she presented the DIANA-LncBase v2 database, that includes more than 70,000 low and high-throughput, (in)direct miRNA:lncRNA experimentally supported interactions, derived from manually curated publications and the analysis of 153 AGO CLIP-Seq libraries. LncBase v2 also hosts in silico predictions of miRNA targets on lncRNAs, identified with the DIANA-microT algorithm, and enables users to easily identify interactions in 66 different cell types, spanning 36 tissues for human and mouse.

The lectures given by Christophe Blanchet, Alexander Goesmann and Rafael Jimenez, as well as the special lecture given by Graziano Pesole, were devoted to the new National networks/infrastructures for bioinformatics which are being developed in the context of the European Strategic Forum on Research Infrastructures (ESFRI). Christophe Blanchet, who has been involved with distributed computing for life sciences since 2001, is currently working at the French Institute of Bioinformatics (IFB) as leader of the e-infrastructure team. Many research infrastructures are now available, producing huge quantities of data, which require many public reference databases and software tools for their analysis. In his talk, whose title was “Bioinformatics Cloud Services for Life Sciences”, Blanchet reported on the activity of IFB, which aims to provide scientists with bioinformatics services relying on the required computing and storage capacities, while providing a user-friendly solution. A selection of bioinformatics software and pipelines have been integrated as turnkey cloud services, now available to scientists.

Alexander Goesmann is professor of Bioinformatics and Systems Biology at Justus-Liebig-University Giessen. In his talk on de.NBI, the German Network for Bioinformatics Infrastructure, he introduced its architecture and illustrated the research topics of its eight service centres. He also presented the BiGi Bioinformatics Resource Center, a joint facility of Bielefeld and Gießen Universities for microbial genome research.

Rafael Jimenez is Chief Technical Officer of ELIXIR, the European Bioinformatics Research Infrastructure. In his talk on “Implementation of the European life-science infrastructure for biological information“, he explained how ELIXIR has started and how important it is for ELIXIR to collaborate with communities and other research infrastructures in order to build effective and coordinated services for users.

Graziano Pesole, finally, presented the state of the art of ELIXIR-ITA, the Italian node of ELIXIR, whose current activities include the provision of support and data to the European node, the support of some established bioinformatics tools of excellence provided by Italian researchers, and the setting up of a training courses. 

### Two-day hands-on tutorials

Four tutorials were held in parallel on 12–13 October, hosted by the Department of Computer Science of the University of Bari. The computational services were exploited on the resources made available by the ReCaS Project in collaboration with the “Istituto di Fisica Nucleare” (INFN) of Bari. Tutorials were focused on cutting-edge methods and approaches to key issues in bioinformatics analysis of omics data.

Fabio Iannelli, from the IFOM – The FIRC Institute of Molecular Oncology (Milan), and Anna De Grassi, from the Department of Bioscience, Biotechnology and Biopharmaceuticals, University of Bari, focused their tutorial on experimental design and bioinformatics approaches to the analysis of genome re-sequencing data for the detection of genomic variations and the identification of candidate variants in cancer and in rare Mendelian disorders. Ioannis Vlachos, from the DIANA Lab, focused his tutorial on the design and analysis of small RNA-seq experiments with a particular attention on crucial aspects such as study design, sample quality check, sequencing instruments and methodologies, data generation/pre-processing and quality control. Methods for genome alignment and/or microRNA expression estimation and online tools for microRNA functional analyses were also examined.

One tutorial devoted to the use of the BioPAX ontology and Pathway Commons dataset in research projects, focusing on network applications was run by Emek Demir. The last tutorial, sponsored by the Italian Flagship project InterOmics, was run by Pasqualina D’Ursi, from the CNR Institute for Biomedical Technologies of Milan. It was focused on applications of fast docking protocols combined with molecular dynamics techniques to predict reliable protein-ligand complexes.

## Introduction to selected papers

### Selection of best papers

Five full papers were already selected for publication in the Journal of Integrative Bioinformatics before the meeting, where they were presented as oral communications. Nine full papers were instead submitted for publication in this Supplement after the workshop. For their evaluation, an Editorial Board was formed by paying attention that topics of submitted manuscripts were properly covered. It included the following Associated Editors:

- José F. Aldana, University of Malaga, Spain
- Ming Chen, Zhejiang University, China
- Domenica D’Elia, Institute for Biomedical Technologies CNR, Italy
- Laurent Falquet, University of Fribourg, Switzerland
- Frédérique Lisacek, Swiss Institute of Bioinformatics, Switzerland
- Sandra Orchard, European Bioinformatics Institute, United Kingdom
- Alfredo Pulvirenti, University of Catania, Italy
- Paolo Romano, IRCCS AOU San Martino IST, Genova, Italy
- Saverio Vicario, Institute for Biomedical Technologies CNR, Italy

Associated Editors managed the reviewing process for one manuscript which was assigned to one of them according to his/her expertise. Two or more referees were selected for each submission: 19 referees were involved overall. Authors were invited to submit an improved version of their manuscript after the reviews were collected. Associated Editors made the final recommendation for each manuscript and, at the end of this process, five papers out of the nine submitted were accepted and included in this Supplement.

Acuña R et al. [5] report about maintaining legacy scientific workflows. They motivated that one major aspect of proprietary, scripted workflows is its structural re-engineering. As concrete implementation, the Workflow Instrumentation for Structure Extraction (WISE) method, that automatically produces structural skeleton from python scripted workflows, was presented. Using this method, the authors demonstrate and discuss the applicability of WISE on several scientific workflows.

The manuscript “Machine Learning approach to discriminate Saccharomyces cerevisiae yeast cells using sophisticated image features” [6] by Tleis M et al., describes image analysis platforms to support the study of yeast cells. The authors set the focus on the automatic extraction of relevant cell features from microscope images and on their classification regarding their discriminating power for morphological cell properties. Results show a significant ability to discriminate different cell strains and conditions. Furthermore, they reveal the benefits of the features-based classification model.

In their work “RetroMine, or how to provide in-depth retrospective studies from Medline in a glance: the hepcidin use-case” [7], de Cadeville et al. describe a text mining approach to perform retrospective studies from Medline. They propose to incorporate the temporal dimension of published events into process of information extraction. This promising approach allows to identify highly relevant biological entities and events published over time among irrelevant background information. A case study regarding hepcidin gene publications over a decade is used to demonstrate the benefits of the proposed filter.

The contribution “OpenLabNotes - An Electronic Laboratory Notebook Extension for OpenLabFramework”, by List et al. [8], introduces an open source Electronic Laboratory Notebook (ELN) with special focus on data protection. To serve as legal document archive, ELNs must prevent scientific frauds through technical means, such as digital signatures. The authors present OpenLabNotes as a powerful and flexible Laboratory Information Management System (LIMS) that allows protecting the intellectual property of users by providing data protection through digital signatures.

Kiseleva et al., report about extracellular electron transfer by means of Microbial Fuel Cells (MFCs), a promising new technology for cost-effective and sustainable wastewater treatment. Their manuscript titled “Taxonomic and functional metagenomic analysis of anodic communities in two pilot-scale microbial fuel cells treating different industrial wastewaters” [9] provides insights into structural shifts that occur in the transition from electrochemically active robust anodic microbial communities, and their anaerobic digester (AD) sludge inocula, to an MFC microbial community and the metabolic potential of electrochemically active microbial populations with wastewater-treating MFCs.

The paper “Improving the accuracy of high-throughput protein-protein affinity prediction may require better training data” by Dias and Kolaczkowski [10], extends their previous work on the prediction of interactions of proteins with small molecules, DNA/RNA and other proteins [11]. Here, the authors concentrate on the analysis of the factors that might contribute to the poor performance of protein-protein affinity prediction. The work provides a survey of main factors, including X-ray crystal resolution, experimental conditions, such as pH/temperature and binding assay, and, unexpectedly, errors in structure-affinity databases.

The paper “NEArender: an R package for functional interpretation of ‘*omics*’ data via network enrichment analysis” by Jeggari and Alexeyenko [12] introduces an R package able to transform raw “*omics*” features of experimental/clinical samples into matrices describing the same samples in a fast and effective, but still sound, way. The authors demonstrate that the statistical power of the new method is increased not only compared to the differential expression analysis on individual genes, but also to the state-of-the-art gene set enrichment analysis. NEArender score matrices can be used in pipeline integration tasks, including phenotype modelling and disease outcome prediction.

Perron et al., present “In silico prediction of lncRNA function using tissue specific and evolutionary conserved expression” [13]. In this work the authors promote a new method to predict the function of protein coding genes (PCGs) and lncRNAs based on the functional prediction score (FPS) computed from gene co-expression networks from different tissue and species. Results are based on the analysis of 30 human tissues and 9 vertebrates. Networks are mined with a methodology inspired by the rank product algorithm used to identify differentially expressed genes. Using different types of reference data, the authors were able to predict putative new annotations for ﻿thousands of lncRNAs and proteins, ranging from cellular localization to relevance for disease and cancer.﻿

In their article “Network-Based Analysis of Transcriptional Profiles from Chemical Perturbations Experiments” [14], Mulas et al., propose a network-based approach for the analysis of gene expression datasets from chemical perturbation experiments, aiming especially to genes associated with chemical pathways of known carcinogenicity and toxicity profiles. A three step pipeline is executed: i) networks from control and exposed samples are built; ii) modules of tightly connected genes are identified and compared; iii) the analysis of clusters is conducted. The approach is straightforward and sound, and its validity is supported by results. It has clear potential applications in drug discovery and repurposing.

The work “GATK hard filtering: tunable parameters to improve variant calling for next generation sequencing targeted gene panel data” by De Summa et al. [15], aims at analysing hard filtering of the Genome Analysis ToolKit (GATK), a powerful toolkit offering a variety of tools with a primary focus on variant discovery and genotyping, to evaluate how the performance of variant calling can be finely tuned to obtain the best results. Authors focused on the case of targeted sequencing of small set of genes when using a Ion Torrent NGS platform. They studied several standard and non-standard GATK filters to be used for hard-filtering in the context of a targeted gene panel sequencing. The results show that filters could be correctly tuned in relation to coverage and type of alterations. Moreover, it could be useful to test by appropriate simulations the design of amplicon gene panels to gain a priori knowledge of the possible problems in variant calling by GATK.

## Abbreviations

ELN: Electronic laboratory notebook
ESFRI: European Strategic Forum on Research Infrastructures
GATK: Genome Analysis ToolKit
IB: Integrative bioinformatics
ICT: Information and communication technology
IFB: French Institute of Bioinformatics
JIB: Journal of integrative bioinformatics
lncRNA: Long non-coding RNA
MFC: Microbial fuel cell
ncRNA: Non-coding RNA
NETTAB: Network Tools and Applications in Biology
WISE: Workflow Instrumentation for Structure Extraction
